# Development and validation of a nomogram to predict risk of septic cardiomyopathy in the intensive care unit

**DOI:** 10.1038/s41598-024-64965-x

**Published:** 2024-06-19

**Authors:** Peng-fei Sun, Cheng-jian Wang, Ying Du, Yu-Qin Zhan, Pan-pan Shen, Ya-hui Ding

**Affiliations:** 1 The 2nd Clinical Medical College of Zhejiang Chinese Medical University, Hangzhou, Zhejiang China; 2 Heart Center, Department of Cardiovascular Medicine, Zhejiang Provincial People’s Hospital (Affiliated People’s Hospital, Hangzhou Medical College), Hangzhou, Zhejiang China

**Keywords:** Sepsis, Septic cardiomyopathy, Predictive model, Nomogram, Intensive care unit, Cardiovascular diseases, Cardiovascular diseases, Metabolic disorders

## Abstract

The aim of this study was to develop a simple but effective nomogram to predict risk of septic cardiomyopathy (SCM) in the intensive care unit (ICU). We analyzed data from patients who were first admitted to the ICU for sepsis between 2008 and 2019 in the MIMIC-IV database, with no history of heart disease, and divided them into a training cohort and an internal validation cohort at a 7:3 ratio. SCM is defined as sepsis diagnosed in the absence of other cardiac diseases, with echocardiographic evidence of left (or right) ventricular systolic or diastolic dysfunction and a left ventricular ejection fraction (LVEF) of less than 50%. Variables were selected from the training cohort using the Least Absolute Shrinkage and Selection Operator (LASSO) regression to develop an early predictive model for septic cardiomyopathy. A nomogram was constructed using logistic regression analysis and its receiver operating characteristic (ROC) and calibration were evaluated in two cohorts. A total of 1562 patients participated in this study, with 1094 in the training cohort and 468 in the internal validation cohort. SCM occurred in 13.4% (147 individuals) in the training cohort, 16.0% (75 individuals) in the internal validation cohort. After adjusting for various confounding factors, we constructed a nomogram that includes SAPS II, Troponin T, CK-MB index, white blood cell count, and presence of atrial fibrillation. The area under the curve (AUC) for the training cohort was 0.804 (95% CI 0.764–0.844), and the Hosmer–Lemeshow test showed good calibration of the nomogram (P = 0.288). Our nomogram also exhibited good discriminative ability and calibration in the internal validation cohort. Our nomogram demonstrated good potential in identifying patients at increased risk of SCM in the ICU.

## Introduction

Acute organ dysfunction is a leading cause of mortality in patients with sepsis, where the heart is often the most commonly and severely affected organ^1,2^. The myocardial dysfunction that arises due to sepsis is known as septic cardiomyopathy (SCM), which is primarily characterized by a reduction in left ventricular ejection fraction (LVEF) and ventricular dilation^3–5^. This is a severe complication, and studies have reported that patients with septic cardiomyopathy (SCM) have a mortality rate that is 35–50% higher compared to sepsis patients without myocardial injury, and they also have a poorer prognosis^6–11^.

Currently, the treatment of SCM primarily focuses on symptomatic treatment of sepsis. Initial treatment depends on early recognition, etiological treatment, fluid resuscitation, and the use of vasopressors. However, when patients exhibit cardiac dysfunction, excessive fluid resuscitation may lead to increased ventricular filling pressure, which can increase cardiac preload and result in hemodynamic imbalance, further affecting patient prognosis^12–14^. At the same time, cardiac protective treatment for critically ill patients is also necessary. β-Blockers, angiotensin-converting enzyme inhibitors, calcium channel blockers, and statins have potential benefits in improving heart failure^14,15^. Therefore, early identification of high-risk patients for SCM is crucial for guiding subsequent treatment and improving prognosis.

Currently, for the early prediction of the likelihood of SCM, researchers around the world have widely discussed many predictive factors^3,7,13,14,16–18^. The elevation of myocardial markers, such as cardiac troponins, is thought to be closely related to the degree of left ventricular dysfunction, the severity of the illness, and mortality rates^13,19,20^. The literature reports that factors like being male, increased age, having a history of heart failure, coronary artery disease, or diabetes, as well as high lactate levels upon admission to the ICU, have been associated with SCM^10,21^. Moreover, high scores on evaluation scales like the Sequential Organ Failure Assessment (SOFA), Simplified Acute Physiology Score II (SAPS II), and Acute Physiology and Chronic Health Evaluation II (APACHE II), in addition to the use of catecholamines, have a positive correlation with the risk of SCM occurrence^22^. The identification of these predictive factors is of significant importance for clinicians in recognizing patients at high risk for SCM and assessing their possible prognosis.

However, due to the complex mechanisms and rapid progression of septic cardiomyopathy, and the lack of clinical specificity, single factors cannot effectively predict SCM. The establishment of clinical risk predictive models could enable the early and effective identification of high-risk individuals for SCM. Currently, there is no predictive model available for the early identification of high-risk patients for SCM in ICU. Therefore, our aim is to develop a new clinical tool to efficiently predict the risk of SCM occurrence in the ICU, facilitate the early diagnosis of SCM, and provide more information for prevention and targeted interventions.

## Materials and methods

### Database

This study aimed to develop and validate a predictive model by utilizing the Medical Information Mart for Intensive Care-IV (MIMIC-IV) database, which was registered for this purpose. The study utilized version 2.2 of the MIMIC-IV database, which contains relevant health data from over 50,000 patients admitted to the intensive care unit of Beth Israel Deaconess Medical Center between 2008 and 2019. The MIMIC-IV database offers a substantial amount of real clinical data, encompassing information about patients admitted to the ICU of a large tertiary hospital. The dataset comprises vital signs, laboratory indicators, medications, diagnostic codes, imaging reports, length of hospital stay, survival data, and other relevant information. Data extraction can be performed using the Structured Query Language (SQL) language for subsequent analysis. The individuals involved in this study completed a series of courses provided by the National Institutes of Health (NIH) and were granted authorization to access the MIMIC-IV database upon successful completion of the necessary evaluation (Certificate No. 52898099). In this study, we utilized version 2.2 of the database and utilized PostgreSQL v13.0 to retrieve data (http://www.PostgreSQL.org/). As all personal data in the database has been deidentified before analysis, the requirement for institutional review board approval was waived, and patient consent was not required^23,24^.

### Study populations

This retrospective analysis examines adult patients with sepsis who were admitted to the ICU. The data used in this study is sourced from the MIMIC-IV database. The inclusion criteria are as follows:

1. Patients diagnosed with sepsis according to the sepsis-3.0 diagnostic criteria^25^. Infected patients were identified using the International Classification of Diseases diagnosis codes, and those with a Sequential Organ Failure Assessment (SOFA) score ≥ 2 were diagnosed with sepsis.
2. Patients who were admitted to the ICU for the first time.
3. Adult patients aged 18 years and above.
4. Patients who have completed an echocardiogram after ICU admission.
5. Patients with an ICU length of stay exceeding 24 h.
6. Patients with no history of underlying cardiac disease and whose current diagnosis does not include chronic heart failure, ischemic heart disease (including acute myocardial infarction, coronary artery atherosclerotic heart disease), congenital heart disease, other cardiomyopathies (including hypertrophic cardiomyopathy, alcoholic cardiomyopathy, nutritional and metabolic cardiomyopathies), valvular heart disease, chronic pulmonary heart disease, and rheumatic heart disease; these are non-clinical trial patients (specific ICD codes are listed in Supplementary Table S1).

### Outcome measure

The outcome event was defined as the diagnosis of SCM during the stay in the ICU. Based on the inclusion criteria from previous studies^9,10,26–29^ and the guidelines of heart failure^30,31^, SCM is defined as sepsis diagnosed in the absence of other cardiac diseases, with echocardiographic evidence of left (or right) ventricular systolic or diastolic dysfunction and a left ventricular ejection fraction (LVEF) of less than 50%.

### Variable collection

Based on current published research regarding potential early predictors of SCM and the clinical relevance and availability of these factors at the time of admission^3,7,10,13,16–22,32^, we compiled the following data: demographic information (age and gender), disease severity scores within the first 24 h after ICU admission (SOFA and SAPS II), the mean value of vital signs within the first 24 h after ICU admission (heart rate, systolic blood pressure, diastolic blood pressure, mean arterial pressure, respiration rate, temperature, and peripheral oxygen saturation), comorbidities (chronic lung disease, diabetes, atrial fibrillation, hypertension), the mean value of laboratory results within the first 24 h after ICU admission (white blood cell count, hemoglobin, blood urea nitrogen, serum creatinine, alanine aminotransferase, aspartate aminotransferase, lactate, cardiac troponin T, CK-MB index), activation of renal replacement therapy within the first 24 h after ICU admission, initiation of mechanical ventilation within the first 24 h after ICU admission, and the use of vasoactive agents within the first 24 h after ICU admission (dopamine, epinephrine, norepinephrine, dobutamine, vasopressin, phenylephrine and milrinone).

To evaluate the dynamic changes in clinical signs, we used the average real variability (ARV) to assess the blood pressure variability (BPV) within the first 24 h after ICU admission^33–35^. The calculation formula is as follows:

$$ ARV = \frac{1}{N - 1}\mathop \sum \limits_{k = 1}^{N - 1} |BP_{k + 1} - BP_{k} | $$

where N represents the number of valid blood pressure measurements and k represents the order of measurements.

Similarly, we introduced the concept of ARV into routine ICU assessed vital signs such as respiration rate, heart rate, peripheral oxygen saturation, and body temperature. By writing SQL code, we extracted the ARV of systolic blood pressure (SBP), ARV of diastolic blood pressure (DBP), ARV of mean arterial pressure (MBP), ARV of respiration rate, ARV of heart rate, ARV of SpO_2_, and ARV of temperature in the first 24 h after ICU admission.

### Management of missing data

Variables with missing data are common in the MIMIC-IV database, with the amount of missing data for different variables ranging from 0 to 58% (Supplementary Table S2). Out of 1562 patients, 393 individuals (25%) had complete data for all variables in the analysis. However, excluding patients with incomplete data can introduce bias into the study. Therefore, we used multiple imputation (MI), based on 5 replications and a chained equation approach method in the R MI procedure, to account for missing data. There were no significant differences in the distributions of any variables with missing data between the imputation datasets and the observed complete case data (Supplementary Table S2).

### Statistical analysis

We used a random sequence to extract 70% of the data to form the training cohort and the remaining 30% constituted the internal validation cohort. We constructed a nomogram with the training cohort and compared the baseline clinical characteristics of patients with and without SCM in the training cohort. We employed the Shapiro–Wilk test to assess whether variables were normally distributed. For continuously distributed variables with a normal distribution, we reported the mean and standard deviation (SD); for continuously distributed variables with a skewed distribution, we reported the median and interquartile range (IQR); for categorical variables, we reported frequencies and percentages. In the baseline characteristics analysis, we used the *t*-test (for normal distribution), chi-square test or Fisher's exact test (for categorical variables), and Kruskal–Wallis test (for skewed distributions) to compare variables and test for statistical differences between groups.

The Least Absolute Shrinkage and Selection Operator (LASSO) method was employed to select the optimal predictive variables for the construction of nomogram. To avoid overcomplicating the clinical prediction model, the lambda value was set to “1se”. Subsequently, a predictive model was developed using multivariate logistic regression analysis, incorporating the risk factors selected in the LASSO model.

The area under the receiver operating characteristic (ROC) curve (AUC) was used to assess the ability of the nomogram to distinguish SCM and non-SCM patients in the training and internal validation cohorts, with the corresponding 95% CI calculated. Calibration plots and the Hosmer–Lemeshow goodness-of-fit test were used to evaluate the consistency between the actual SCM risk and the probability predicted by the nomogram. Additionally, Decision Curve Analysis (DCA) was performed to determine the clinical usefulness and net benefit at different threshold probabilities.

The sample size for the predictive model was determined by the ratio of outcome events to estimable predictor variables, with a minimum requirement of 10, according to Harrell’s guidelines^36^. Our final model included 5 variables. Therefore, this requires a minimum of 50 positive events. As a study with a larger sample size, in our training cohort, there were 147 positive events, indicating that we have met this requirement.

The statistical analysis was conducted using R software (version 4.1.3; R Foundation for Statistical Computing, Vienna, Austria; https://www.r-project.org). All significance tests were two-sided, with a P-value < 0.05 considered statistically significant.

### Ethics approval and consent to participate

As all personal data in the database has been deidentified before analysis, the requirement for institutional review board approval was waived, and patient consent was not required.

### Consent for publication

Patient consent was not required.

## Results

**Figure 1:**
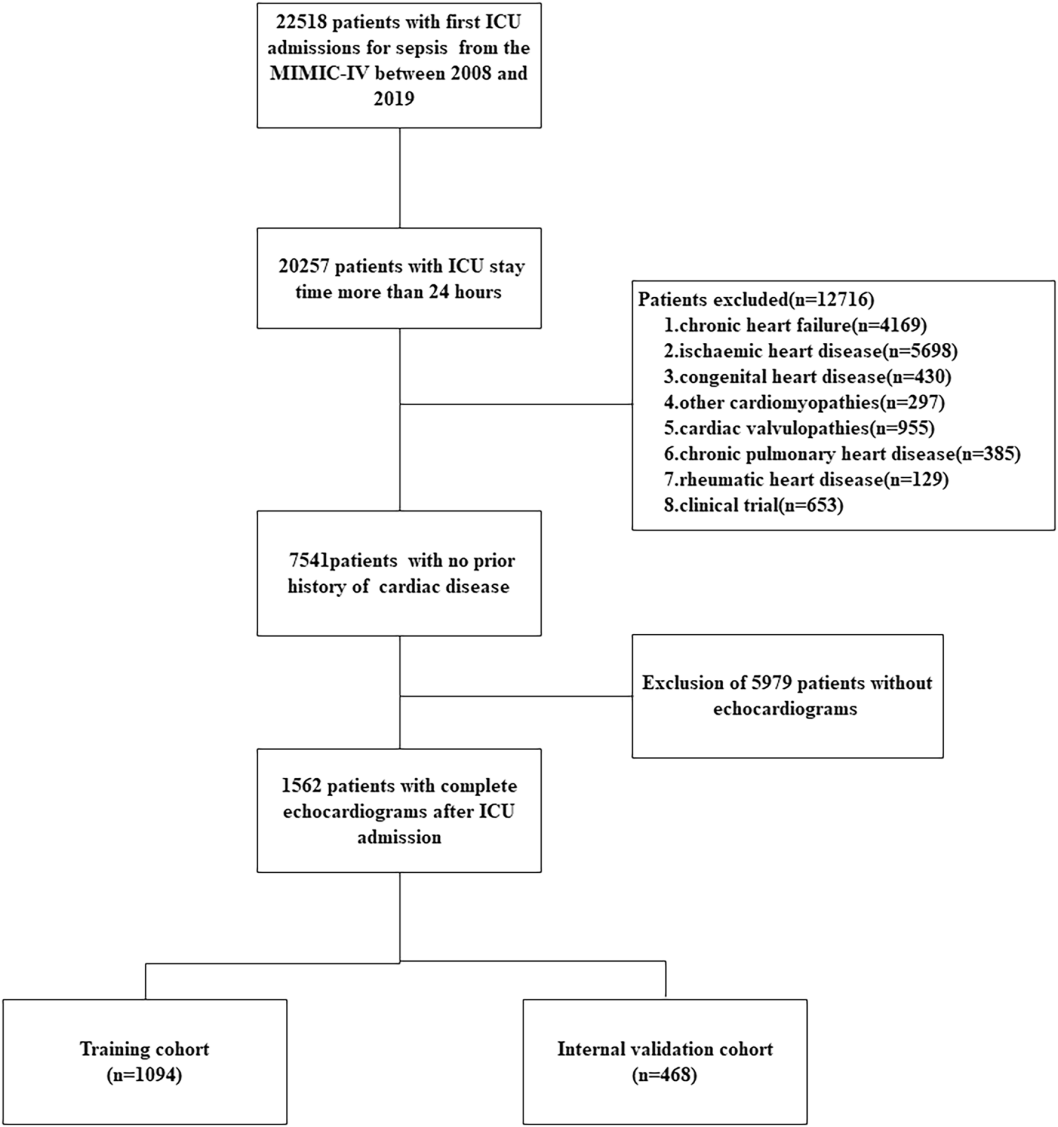
Flowchart showing the process of cohort selection.

Figure 1 shows the process of cohort selection. We enrolled a total of 1562 patients in our study, with 1094 patients in the training cohort, 468 in the internal validation cohort. In the training cohort, the patients had a mean age of 61.1 ± 16.8 years, and 53% (n = 580) were male, and the median score of SAPSII was 37 (28–47). Among these patients, 32.6% (n = 357) received vasoactive drug therapy on the first day, and 13.4% (n = 147) were diagnosed with SCM. In the internal validation cohort, 16.0% (n = 75) of the patients eventually developed SCM. The baseline characteristics of the cohorts are presented in Table 1.

**Table 1:** Baseline characteristics of the population.

Variables	Overall (n = 1562)	Training cohort (n = 1094)	Internal validation cohort (n = 468)	*P*
Male, n (%)	817 (52.3)	580 (53)	237 (50.6)	0.389
Age, year, mean (SD)	61.0 (17.0)	61.1 (16.8)	61.0 (17.5)	0.887
Heart rate, bpm, mean (SD)	89.7 (17.9)	89.5 (17.9)	90.0 (18.0)	0.747
SBP, mmHg, mean (SD)	117 (16.6)	117 (16.8)	117 (16.3)	0.794
DBP, mmHg, mean (SD)	63.4 (10.6)	63.3 (10.4)	63.5 (11.0)	0.637
MBP, mmHg, mean (SD)	77.7 (10.9)	77.7 (10.9)	77.8 (11.0)	0.795
Respiratory rate, mean (SD)	20.7 (4.53)	20.7 (4.56)	20.6 (4.47)	0.769
Temperature, °C, mean (SD)	37.0 (0.69)	37.0 (0.69)	37.0 (0.68)	0.991
SpO_2_, %, mean (SD)	96.7 (2.32)	96.7 (2.25)	96.8 (2.50)	0.345
ARV of 24 h heart rate, mean (SD)	5.37 (2.80)	5.38 (2.96)	5.35 (2.40)	0.806
ARV of 24 h SBP, mean (SD)	11.6 (4.50)	11.5 (4.39)	11.8 (4.74)	0.260
ARV of 24 h DBP, mean (SD)	8.71 (4.29)	8.58 (4.20)	9.00 (4.47)	0.081
ARV of 24 h MBP, mean (SD)	9.20 (4.44)	9.14 (4.51)	9.34 (4.28)	0.399
ARV of 24 h respiratory rate, mean (SD)	3.31 (1.05)	3.33 (1.05)	3.26 (1.04)	0.225
ARV of 24 h temperature, mean (SD)	0.35 (0.15)	0.35 (0.15)	0.35 (0.15)	0.955
ARV of 24 h SpO_2_, mean (SD)	1.49 (0.66)	1.49 (0.66)	1.49 (0.66)	0.897
SAPSII, median (IQR)	37.0 (28.0, 47.0)	37.0 (28.0, 47.0)	37.0 (28.8, 47.3)	0.795
SOFA, median (IQR)	3.0 (2.0, 4.0)	3.0 (2.0, 4.0)	3.0 (2.0, 5.0)	0.196
WBC, (× 10^9^/L), median (IQR)	11.5(7.9, 16.0)	11.4 (7.8, 16.1)	11.8 (8.3, 15.6)	0.580
Hemoglobin, g/dL, median (IQR)	10.6(9.1, 12.4)	10.7 (9.1, 12.4)	10.5(9.1, 12.3)	0.212
BUN, mg/dL, median (IQR)	20.6(13.5, 33.7)	20.6(13.5, 33.7)	20.6(13.5, 33.5)	0.863
Creatine, mg/dL, median (IQR)	1.00 (0.70, 1.67)	1.00 (0.70, 1.63)	1.00 (0.72, 1.72)	0.932
ALT, U/L, median (IQR)	31.0(18.5, 69.0)	31.0 (18.0, 69.2)	32.0(20.0, 68.6)	0.245
AST, U/L, median (IQR)	46.13 (27.0, 106.3)	46.0(26.5, 104.1)	51.2(28.0, 125.0)	0.071
Lactate, mmol/L, median (IQR)	1.68 (1.20, 2.40)	1.65 (1.20, 2.40)	1.70 (1.19, 2.42)	0.918
Troponin T, ng/mL, n (%)				0.421
< 0.01	997 (63.8)	712 (65.1)	285 (60.9)	
≥ 0.01 and < 0.1	426 (27.3)	288 (26.3)	138 (29.5)	
≥ 0.1 and < 1	106 ( 6.8)	73 (6.7)	33 (7.1)	
≥ 1	33 ( 2.1)	21 (1.9)	12 (2.6)	
CK-MB index, median (IQR)	0.014 (0.008, 0.026)	0.014 (0.008, 0.026)	0.014 (0.008, 0.026)	0.104
Chronic pulmonary disease, n (%)	342 (21.9)	253 (23.1)	89 (19)	0.072
Diabetes, n (%)	397 (25.4)	270 (24.7)	127 (27.1)	0.307
Atrial fibrillation, n (%)	367 (23.5)	253 (23.1)	114 (24.4)	0.599
Hypertension, n (%)	836 (53.5)	579 (52.9)	257 (54.9)	0.47
Mechanical ventilation within the first 24 h in ICU, n (%)	1064 (68.1)	738 (67.5)	326 (69.7)	0.393
The usage of the vasoactive agent in the first 24 h, n (%)	516 (33.0)	357 (32.6)	159 (34)	0.606
Dialysis activated within 24 h in ICU, n (%)	95 ( 6.1)	64 (5.9)	31 (6.6)	0.558
SCM, n (%)	222 (14.2)	147 (13.4)	75 (16)	0.180

*SD* standard deviation, *IQR* interquartile range, *SCM* septic cardiomyopathy, *SBP s*ystolic blood pressure, *DBP* diastolic blood pressure, *MBP* mean arterial pressure, *SpO*_*2*_ percutaneous arterial oxygen saturation, *ARV* average real variability, *SAPS* simplified acute physiology score, *SOFA* sequential organ failure assessment, *WBC* white blood cell, *BUN* blood urea nitrogen, *ALT* alanine aminotransferase, *AST* aspartate aminotransferase, *CK-MB* creatine kinase isoenzyme-MB, *ICU* intensive care unit.

**Figure 2:**
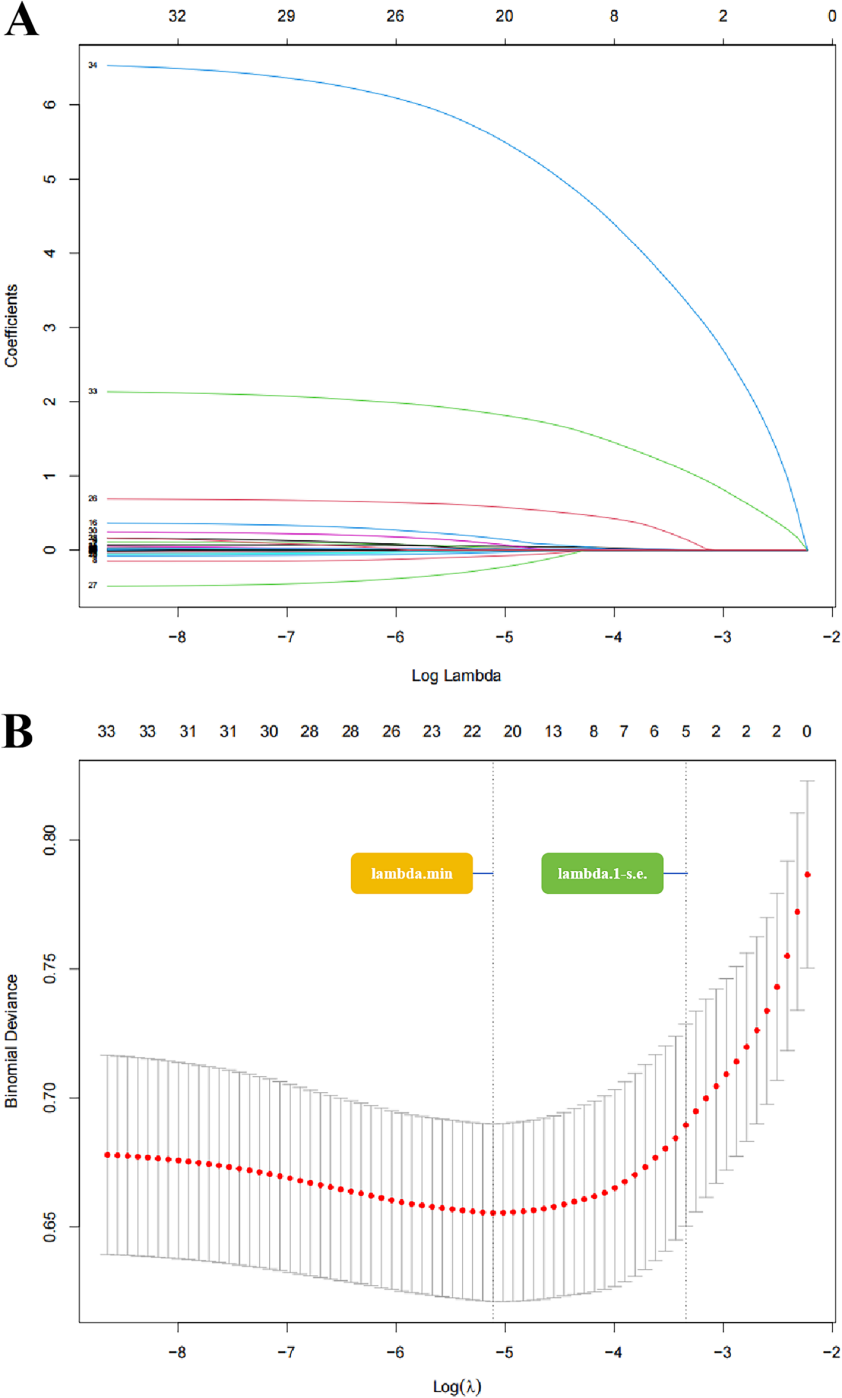
(**A**) LASSO coefficient profiles of the 34 risk factors. (**B**) Five risk factors selected using LASSO Cox regression analysis. The two dotted vertical lines were drawn at the optimal scores by minimum criteria and 1-s.e. criteria (At minimum criteria including age, heart rate, SBP, respiratory rate, temperature, SpO_2_, ARV of 24 h heart rate, ARV of 24 h DBP, ARV of 24 h SpO_2_, hemoglobin, WBC, BUN, ALT, atrial fibrillation, hypertension, SAPSII, SOFA, Mechanical ventilation within the first 24 h in ICU, Troponin T, CK-MB index; At 1-s.e. criteria including WBC, SAPSII, atrial fibrillation, Troponin T, CK-MB index).

We investigated the predictive factors for SCM in the training cohort and observed significant differences between the SCM group and the non-SCM group. Compared to the non-SCM group, the SCM group had higher ages, heart rates, respiratory rates, ARV of 24 h SpO_2_, ARV of 24 h DBP, white blood cell counts, BUN, and creatinine levels, while SBP, DBP, and MBP were lower. Additionally, significant associations were found between SCM and troponin T, CK-MB index, SAPS II scores, the administration of vasoactive drugs on the first day, and the coexistence of atrial fibrillation (Table 2). Using LASSO regression, five variables were selected: Troponin T, CK-MB index, SAPS II scores, WBC count, and the coexistence of atrial fibrillation. Figure 2 illustrates the variable selection process. Based on the rules of the prediction model and clinical applicability, we hope to achieve sufficient fitting with as few parameters as possible. Therefore, we identified the five variables as optimal predictors. These five variables were included in the multivariable logistic regression model to predict the occurrence of SCM, and their odds ratios and 95% confidence intervals (CI) are shown in Table 3.

**Table 2:** Univariate analysis of septic cardiomyopathy in the training cohort.

Variables	Overall (n = 1094)	Non-SCM (n = 947)	SCM (n = 147)	*P*
Male, n (%)	514 (47.0)	437 (46.1)	77 (52.4)	0.159
Age, year, mean (SD)	61.0 (16.8)	60.3 (16.5)	65.6 (18.4)	< 0.001
Heart rate, bpm, mean (SD)	89.6 (17.9)	89.0 (17.8)	92.7 (18.5)	0.021
SBP, mmHg, mean (SD)	117 (16.8)	118 (16.7)	114 (17.0)	0.004
DBP, mmHg, mean (SD)	63.3 (10.3)	63.6 (10.3)	61.4 (10.5)	0.016
MBP, mmHg, mean (SD)	77.7 (10.9)	78.0 (10.9)	75.4 (10.5)	0.008
Respiratory rate, mean (SD)	20.7 (4.56)	20.5 (4.47)	21.9 (4.94)	< 0.001
Temperature, °C, mean (SD)	37.0 (0.69)	37.0 (0.66)	36.9 (0.86)	0.029
SpO_2_, %, mean (SD)	96.7 (2.25)	96.7 (2.21)	96.6 (2.49)	0.719
ARV of 24 h heart rate, mean (SD)	5.39 (2.96)	5.43 (2.99)	5.12 (2.74)	0.242
ARV of 24 h SBP, mean (SD)	11.5 (4.39)	11.5 (4.36)	11.7 (4.62)	0.543
ARV of 24 h DBP, mean (SD)	8.58 (4.20)	8.49 (3.95)	9.19 (5.50)	0.061
ARV of 24 h MBP, mean (SD)	9.14 (4.51)	9.08 (4.58)	9.53 (4.01)	0.271
ARV of 24 h respiratory rate, mean (SD)	3.33 (1.06)	3.33 (1.03)	3.34 (1.19)	0.953
ARV of 24 h temperature, mean (SD)	0.35 (0.15)	0.35 (0.16)	0.35 (0.15)	0.76
ARV of 24 h SpO_2_, mean (SD)	1.49 (0.67)	1.48 (0.62)	1.60 (0.89)	0.035
SAPSII, median (IQR)	37.0 (28.0, 47.0)	37.0 (27.0, 46.0)	41.0 (35.0, 55.5)	< 0.001
SOFA, median (IQR)	3.0 (2.0, 4.0)	3.0 (2.0, 4.0)	3.0 (2.0, 5.0)	0.056
WBC, (× 10^9^/L), median (IQR)	11.4 (7.75, 16.10)	11.0 (7.50, 15.72)	13.1 (9.22, 19.19)	< 0.001
Hemoglobin, g/dL, median (IQR)	10.7 (9.18, 12.45)	10.7 (9.21, 12.45)	10.4 (9.03, 12.43)	0.502
BUN, mg/dL, median (IQR)	20.6 (13.53, 33.65)	19.8 (13.00, 32.90)	29.0 (17.00, 40.75)	< 0.001
Creatine, mg/dL, median (IQR)	1.0 (0.70, 1.64)	1.0 (0.70, 1.55)	1.2 (0.85, 2.02)	0.048
ALT, U/L, median (IQR)	31.0 (18.00, 68.88)	31.0 (17.75, 63.50)	35.0 (20.50, 87.00)	0.212
AST, U/L, median (IQR)	44.0 (26.00, 101.88)	44.0 (25.50, 97.00)	48.0 (29.00, 121.50)	0.296
Lactate, mmol/L, median (IQR)	1.7 (1.20, 2.40)	1.7 (1.20, 2.37)	1.8 (1.30, 2.70)	0.176
Troponin T, ng/mL, n (%)				< 0.001
< 0.01	712 (65.1)	659 (69.6)	53 (36.1)	
≥ 0.01 and < 0.1	288 (26.3)	231 (24.4)	57 (38.8)	
≥ 0.1 and < 1	73 ( 6.7)	54 (5.7)	19 (12.9)	
≥ 1	21 ( 1.9)	3 (0.3)	18 (12.2)	
CK-MB index, median (IQR)	0.014 (0.008, 0.026)	0.013 (0.008, 0.017)	0.038 (0.013, 0.074)	< 0.001
Chronic pulmonary disease, n (%)	253 (23.1)	213 (22.5)	40 (27.2)	0.207
Diabetes, n (%)	270 (24.7)	226 (23.9)	44 (29.9)	0.112
Atrial fibrillation, n (%)	253 (23.1)	197 (20.8)	56 (38.1)	< 0.001
Hypertension, n (%)	579 (52.9)	507 (53.5)	72 (49)	0.303
Mechanical ventilation within the first 24 h in ICU, n (%)	1064 (68.1)	738 (67.5)	627 (66.2)	0.025
The usage of the vasoactive agent in the first 24 h, n (%)	357 (32.6)	294 (31)	63 (42.9)	0.004
Dialysis activated within 24 h in ICU, n (%)	1030 (94.1)	896 (94.6)	134 (91.2)	0.096

*SD* standard deviation, *IQR* interquartile range, *SCM* septic cardiomyopathy, *SBP s*ystolic blood pressure, *DBP* diastolic blood pressure, *MBP* mean arterial pressure, *SpO*_*2*_ percutaneous arterial oxygen saturation, *ARV* average real variability, *SAPS* simplified acute physiology score, *SOFA* sequential organ failure assessment, *WBC* White blood cell, *BUN* blood urea nitrogen, *ALT* alanine aminotransferase, *AST* aspartate aminotransferase, *CK-MB* creatine kinase isoenzyme-MB, *ICU* intensive care unit.

**Table 3:** Multivariate regression analysis for the prediction of septic cardiomyopathy in training cohort.

Predictors	Estimate	Adjusted OR (95% CI)	*P*
(Intercept)	− 3.962		
SAPSII	0.015	1.01 (1–1.03)	0.033
Troponin T (ng/mL)			
< 0.01	0	1 (Ref)	
≥ 0.01 and < 0.1	1.054	2.87 (1.85–4.44)	< 0.001
≥ 0.1 and < 1	1.25	3.49 (1.85–6.57)	< 0.001
≥ 1	3.999	54.57 (14.82–200.91)	< 0.001
CK-MB index	6.595	731.58 (51.76–10,339.54)	< 0.001
WBC	0.028	1.03 (1.01–1.05)	0.002
Atrial fibrillation	0.735	2.08 (1.37–3.16)	0.001

*SAPS* simplified acute physiology score. *CK-MB* creatine kinase isoenzyme-MB. *WBC* White blood cell.

**Figure 3:**
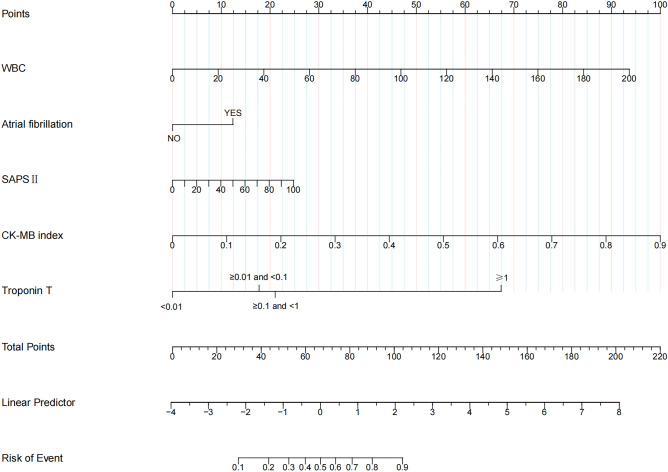
Nomogram to calculate the risk score and predict the probability of SCM. By tracing a vertical ascent from the values of each of the five predictors in the chart to intersect with the “Points” line above, the cumulative score derived from these points can be aligned within the “Total points” section. This aggregate score can then be projected horizontally to intercept the “Risk of Event” axis to predict the risk of SCM.

The SCM prediction probabilities based on regression coefficients can be used with the formula $\frac{1}{{1 + e^{ - LP} }}$, where LP (linear predictor) is equal to − 3.962 + 0.015 × SAPSII + 1.054 × Troponin T ≥ 0.01 and < 0.1 + 1.25 × Troponin T ≥ 0.1 and < 1 + 3.999 × Troponin T ≥ 1 + 6.595 × CK-MB index + 0.028 × WBC + 0.735 × Atrial fibrillation. Finally, the nomogram prediction mode is drawn based on R software (Fig. 3). The nomogram prediction model was constructed based on a weighted analysis of the regression coefficients of risk factors. The scores of each risk factor were calculated individually and the total scores were calculated. The corresponding value of the total score was the predicted probability of SCM.

**Figure 4:**
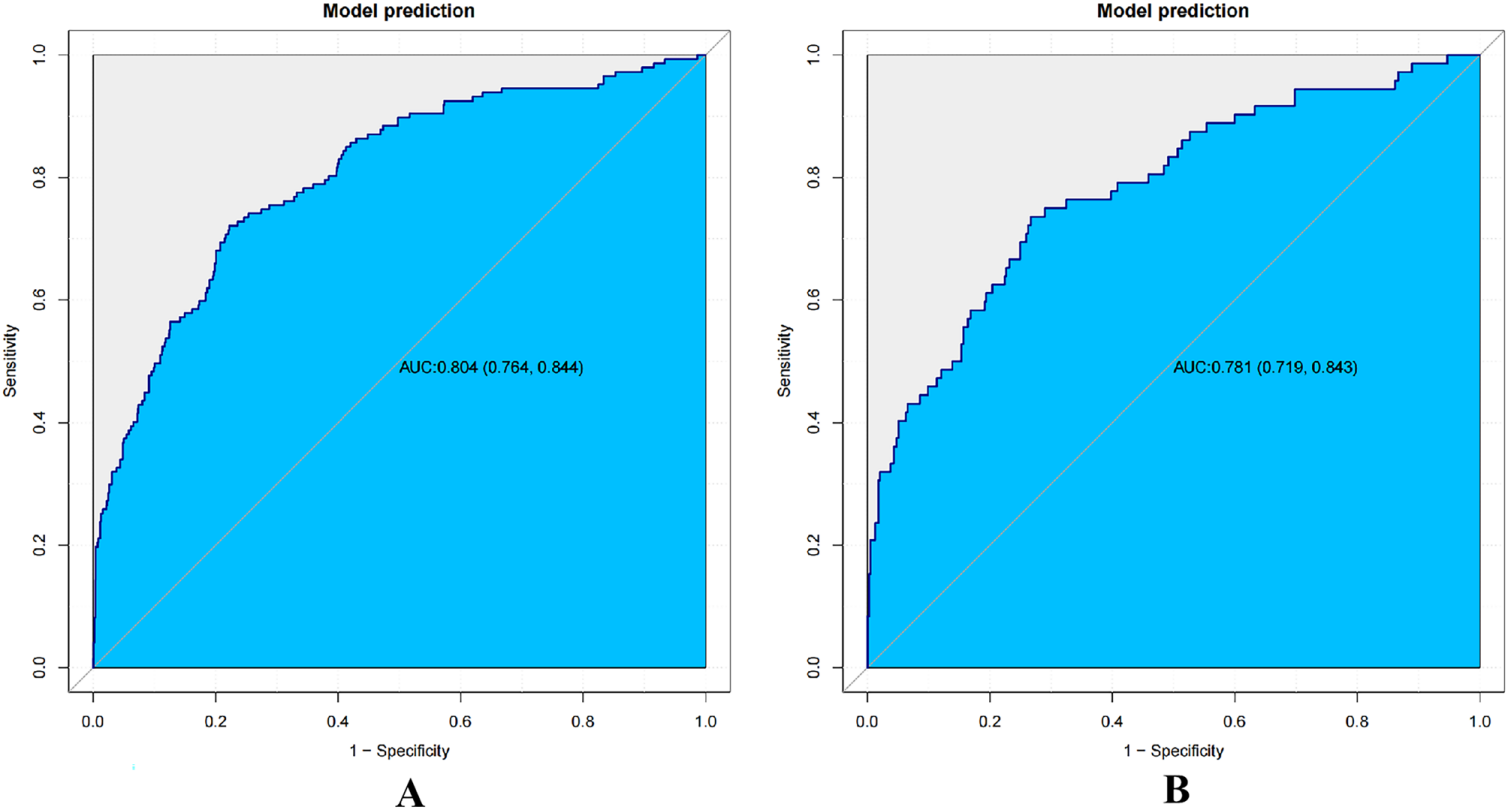
ROC curve of SCM in training (**A**) and internal validation cohort (**B**). The model's capacity to distinguish between SCM was assessed using the ROC curve. In the training cohort (**A**), the AUC of our nomogram was 0.804 (95% CI 0.764–0.844). In the internal validation cohort (**B**), the AUC of our nomogram was 0.781 (95% CI 0.719–0.843).

**Figure 5:**
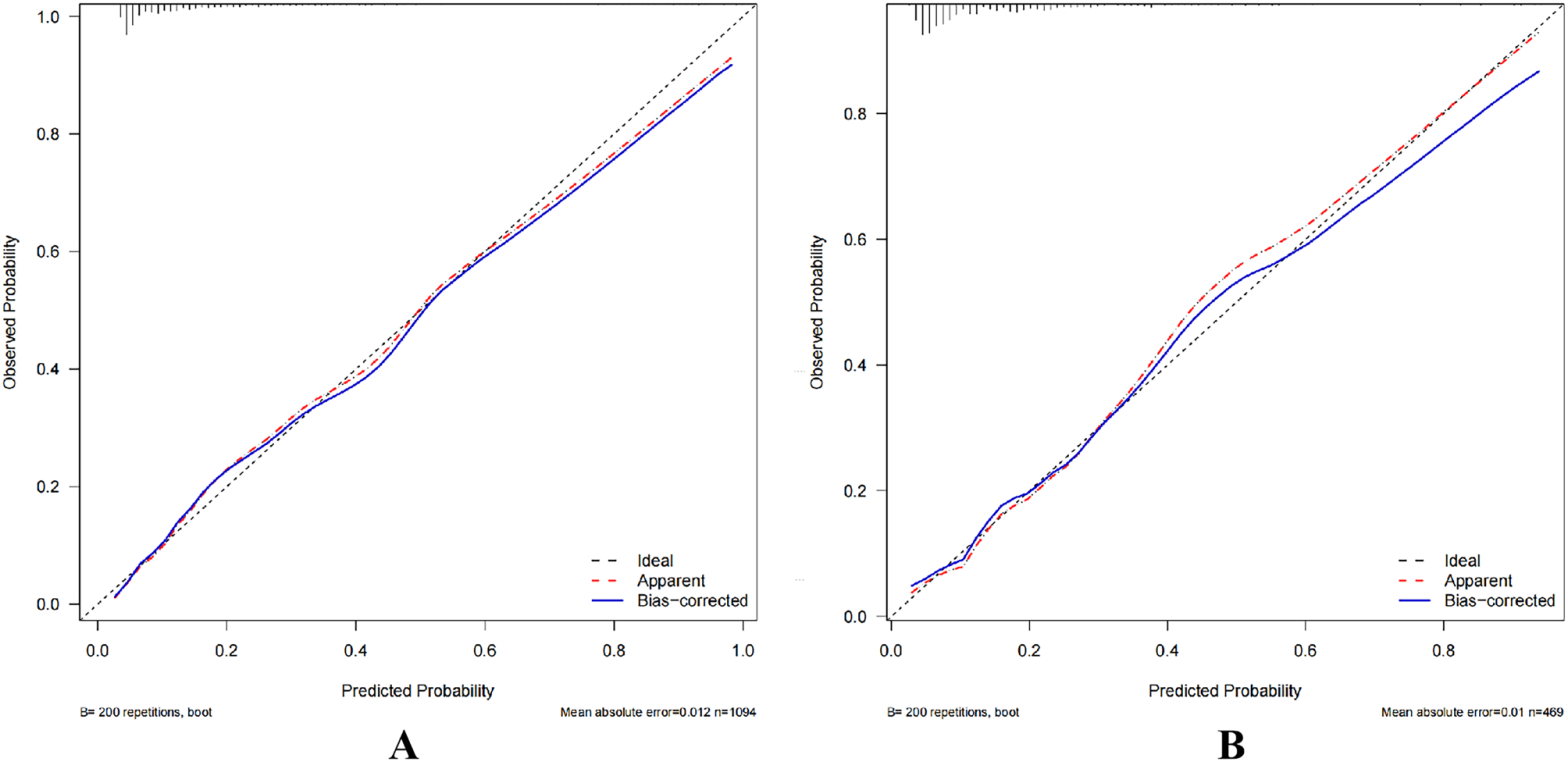
Calibration plot for the nomogram in training (**A**) and internal validation cohort (**B**). Ideal is the ideal reference line for the nomogram. Apparent represents the performance of the nomogram, and Bias-corrected represents the performance when bias in the nomogram is corrected.

The nomogram’s discrimination ability was assessed using the receiver operating characteristic (ROC) curve, with an area under the curve (AUC) of 0.804 (95% CI 0.764–0.844) for the training cohorts, 0.781 (95% CI 0.719–0.843) for the internal validation cohorts. In the training cohort, the sensitivity under the maximum Youden index of the ROC curve was 77.8% and the specificity was 72.1%. In the internal validation cohort, the corresponding values were 73.3% and 73.6% (Fig. 4). Hosmer’s test demonstrated a good fit of the model to the data (χ^2^ = 9.288, P = 0.288), and calibration graph for the nomogram displayed favorable agreement between predicted and actual SCM risk (Fig. 5).

**Figure 6:**
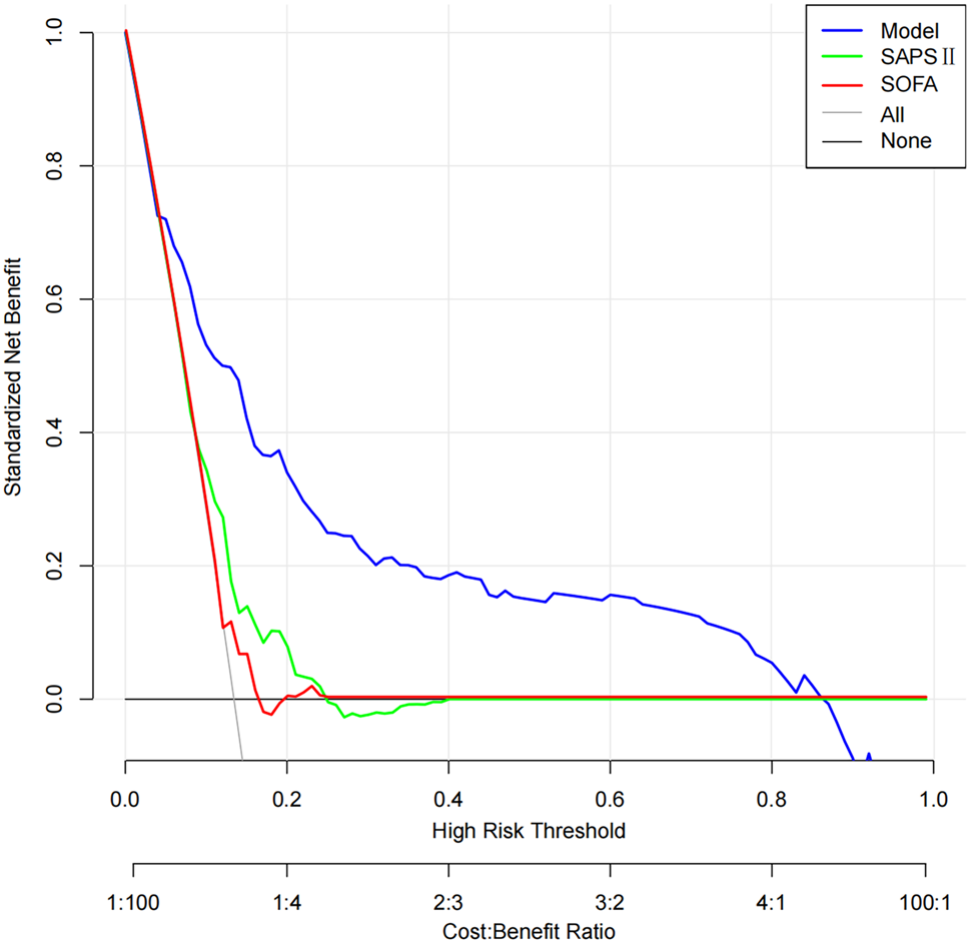
Decision curve analysis (DCA) plots compare the clinical impact of our nomogram and the SOFA and SAPS II scales in the training cohort. The DCA plot shows the standard net benefit in predicting SCM. Across a threshold probability range of 5–87%, our nomogram demonstrates stable clinical net benefits in training cohort, with higher clinical benefits for high SCM risk patients compared to o the SOFA and SAPS II scales.

In addition, we also employed the Decision Curve Analysis (DCA) to compare the clinical impact of the SOFA and SAPS II scales with our nomogram (Fig. 6). The results suggest that our nomogram yields a higher net benefit across a threshold probability range of 5–87% when compared to the SOFA and SAPS II scales.

## Discussion

In this study, we conducted a retrospective analysis of data from 1562 patients admitted to the ICU due to sepsis. We examined dynamic parameters of vital sign variability over time, characteristic biomarkers, common comorbidities, and their relationships with SCM. These parameters were incorporated into a nomogram to predict the likelihood of SCM occurrence in the ICU, aiding in the early identification of patients at high risk for SCM. To our knowledge, this is the first study to develop a predictive model for SCM in ICU patients with sepsis. To facilitate clinical use, we included parameters in the nomogram that are easy to obtain and evaluate, such as Troponin T levels, CK-MB index, SAPS II score, white blood cell count, and the presence of atrial fibrillation. The model demonstrated good discriminative ability and calibration in both the training cohort and an internal validation cohort, confirming its reliability.

Troponin is a highly sensitive and specific protein biomarker of myocardial injury, commonly used in diagnosing Acute Coronary Syndrome (ACS). Elevated troponin levels are often seen in patients with septic shock. Research indicates that between 43 and 85% of patients with sepsis exhibit elevated cardiac troponin^37–39^. Elevated levels of troponin during sepsis may reflect changes in the permeability of myocardial cells or necrosis of myocardial cells due to vascular injury^40^. In patients with sepsis, elevated levels of Troponin) are associated with the degree of left ventricular dysfunction, the severity of the condition, and mortality rates^13,19,20^. It is an independent risk factor for mortality in patients with sepsis^41^. Therefore, our study incorporates troponin T into the nomogram of SCM and stratifies scores based on the value of troponin, balancing simplicity required in clinical settings without compromising discrimination. Results show that relative to a troponin-negative status, higher troponin levels are associated with an increased risk of SCM. However, patients in the ICU often present with complex conditions, including multiple organ failures, such as renal impairment which can affect troponin levels. Recognizing that using troponin alone may not offer high specificity and sensitivity in predict SCM, it is necessary to combine it with CK-MB to enhance the diagnostic accuracy in patients with sepsis. CK-MB is primarily found in the heart muscle and is released into the bloodstream when myocardial cells are damaged or injured, making it a specific marker for myocardial damage. The CK-MB index, typically the ratio of CK-MB to total Creatine Kinase, has been shown by some studies to suggest significant myocardial damage when the CK-MB index ranges between 5 to 30%^42^. Our analysis indicates a significant correlation between the CK-MB index and SCM, suggesting that the CK-MB index can be considered a potential biomarker for assessing the extent of cardiac damage in patients with sepsis.

White blood cells are a traditional inflammatory marker frequently used to define sepsis and assess the severity of the inflammatory response, with elevated counts typically indicating an intensified inflammatory response^43^. The fundamental mechanism of SCM involves the activation of monocytes and macrophages triggered by infectious stimuli, which release various local and systemic inflammatory mediators^44–49^. This intense inflammatory response can disrupt the homeostasis of the cardiovascular system, potentially leading to septic shock. One of the manifestations of cardiovascular dysfunction caused by septic shock is myocardial depression^8^, which is a characteristic feature of SCM.

In addition to validating already reported risk factors, we have found a significant correlation between concomitant atrial fibrillation and SCM. Previous studies have identified sepsis as an independent risk factor for new-onset atrial fibrillation^50^. In the context of sepsis, levels of inflammatory response within the body are elevated, particularly tumor necrosis factor-alpha and interleukin-1 beta^46–49^. These cytokines not only directly affect myocardial cells by inhibiting their contractile function but their synergistic interaction further amplifies the damage to the myocardium. In a high-inflammatory state, when combined with aggressive fluid resuscitation, this can lead to an acute rise in left ventricular end-diastolic pressure and left atrial dilation, increasing the risk of atrial fibrillation. The arrhythmia induced by atrial fibrillation can cause fluctuations in cardiac output and irregularities in ventricular contractions, affecting the efficiency of the heart as a pump and potentially leading to insufficient myocardial oxygen supply. Additionally, prolonged atrial fibrillation may cause structural changes in the left atrium and may have a long-term impact on cardiac conduction and pumping function. The interaction of these various factors not only exacerbates the impact of sepsis on myocardial function but may ultimately contribute to the development of SCM.

The factors influencing the occurrence of SCM are numerous, and in ICU, the vital signs of patients with sepsis change rapidly. Therefore, understanding the dynamic changes in a patient's vital signs over time is important for predicting SCM. We recognize that vital signs can be reflected to some extent using means and extreme values. However, this overlooks the dynamic changes in clinical features of patients, resulting in the loss of data characteristics. Hence, based on the temporal nature of data in the MIMIC database and associated literature, we used the Average Real Variability (ARV) to describe blood pressure variability. ARV takes into account the sequence of measurements and reflects the average absolute change between continuous blood pressure readings, correcting the limitation that the standard deviation only reflects the dispersion of blood pressure values around the mean. Research indicates that the ARV index can more reliably reflect the variability of a time series than SD can^34,35^. Current studies show a clear correlation between ARV of DBP in the first 24 h in ICU patients with acute myocardial infarction and an increased risk of short-term and long-term mortality^35^. Our study is the first to utilize ARV to evaluate blood pressure variability within the first 24 h after a patient’s admission to the ICU and to introduce the concept of ARV to other routinely assessed vital signs in ICU such as respiratory rate, heart rate, oxygen saturation, and body temperature. Our research found that ARV of 24 h SpO_2_ and ARV of 24 h DBP may be correlated with the occurrence of SCM. However, based on the rules of the prediction model and clinical applicability, we hope to achieve sufficient fitting with as few parameters as possible. Therefore, these were not included in the final model. Nevertheless, we acknowledge their potential in identifying high-risk SCM patients in the ICU and aim to further validate and optimize the precision and reliability of this tool through subsequent research.

Furthermore, Decision Curve Analysis offers a robust method for assessing the clinical utility of predictive models. By comparing the net benefit of our nomogram to that of SOFA and SAPS II scales across different threshold probabilities, the DCA curve indicates that our nomogram provides greater clinical decision-making benefits. However, additional validation studies are necessary to confirm the generalizability and reliability of our nomogram across different populations and settings.

Nonetheless, our study does have some limitations. Firstly, it is a single-center retrospective study that utilizes data collected over a relatively long period. As medications, devices, and diagnostic reagents evolve over time, these developments could impact the accuracy of the results. Secondly, while including concise and objective predictive factors facilitates clinical application, the limited parameters might restrict the predictive performance of our nomogram. Thirdly, missing data were handled using multiple imputation techniques, which might decrease the accuracy of the final model. Fourthly, our model predicts the likelihood of SCM in ICU patients with sepsis, but its applicability in general wards requires further study. Fifthly, since the diagnosis of SCM heavily relies on echocardiography, the interpretation of echocardiography results is somewhat subjective, depending on the examiner's skill level and experience, which could affect the extrapolation of the results. Lastly, this study only underwent internal validation. The discriminative ability of the model needs further validation in external cohorts to enhance its generalizability.

## Conclusion

This study indicates that Troponin T, CK-MB index, SAPS II score, WBC count, and concurrent atrial fibrillation can serve as early predictive indicators for SCM in the ICU. Simultaneously, we have developed a simple and effective predictive model to identify high-risk SCM patients in the ICU, demonstrating good discriminative ability and calibration.

### Supplementary Information

Supplementary Table S1.

Supplementary Table S2.

## Data Availability

The datasets analyzed in this study are publicly available summary statistics. Data used can be obtained upon a reasonable request to the corresponding author.

## Abbreviations

AUC: Area under the curve
ALT: Alanine aminotransferase
AST: Aspartate aminotransferase
BUN: Blood urea nitrogen
CI: Confidence interval
DCA: Decision curve analysis
ICU: Intensive care unit
IQR: Interquartile range
LASSO: The Least Absolute Shrinkage and Selection Operator
LVEF: Left ventricular ejection fraction
MIMIC: Medical Information Mart for Intensive Care
OR: Odds ratio
ROC: Receiver operating characteristic
SCM: Septic cardiomyopathy
SD: Standard deviation
SOFA: Sequential Organ Failure Assessment
SBP: Systolic blood pressure
DBP: Diastolic blood pressure
MBP: Mean arterial pressure
SpO_2_: Percutaneous arterial oxygen saturation
ARV: Average real variability
SAPS: Simplified acute physiology score
WBC: White blood cell
BUN: Blood urea nitrogen
CK-MB: Creatine kinase isoenzyme-MB
