# Supplementary material for: Development and validation of a nomogram to predict risk of septic cardiomyopathy in the intensive care unit
Source: Sci Rep. 2024 Jun 19;14:14114. doi: 10.1038/s41598-024-64965-x (PMC11187202; doi:10.1038/s41598-024-64965-x)
Supplement: Supplementary file 1 — Supplementary Table S1. [file 41598_2024_64965_MOESM1_ESM.docx]

**Table S1** ICD codes or SQL queries for obtaining ICD codes

| Disease | ICD9 | ICD10 |
| --- | --- | --- |
| Chronic heart failure | '42822','42823','42832','42833','42842','40201','40211','40291','40401','40403','40413','40491','40493','40411' | 'I5022','I5023','I5032','I5033','I5042','I5043','I50812','I50813','I110','I130','I132', |
| Ischemic heart disease | '41000','41001','41002','41010','41011','41012','41020','41021','41022','41030','41031','41032','41040','41041','41042','41050','41051','41052','41060','41061','41062','41070','41071','41072','41080','41081','41082','41090','41091','41092','4110','4111','41181','41189','412','4130','4131','4139','41400','41401','41402','41403','41404','41405','41406','41407','41410','41411','41412','41419','4142','4143','4144','4148','4149' | 'I20','I200','I201','I208','I209','I21','I210','I2101','I2102','I2109','I211','I2111','I2119','I212','I2121','I2129','I213','I214','I219','I21A','I21A1','I21A9','I22','I220','I221','I222','I228','I229','I23','I230','I231','I232','I233','I234','I235','I236','I237','I238','I24','I240','I241','I248','I249','I25','I251','I2510','I2511','I25110','I25111','I25118','I25119','I252','I253','I254','I2541','I2542','I255','I256','I257','I2570','I25700','I25701','I25708','I25709','I2571','I25710','I25711','I25718','I25719','I2572','I25720','I25721','I25728','I25729','I2573','I25730','I25731','I25738','I25739','I2575','I25750','I25751','I25758','I25759','I2576','I25760','I25761','I25768','I25769','I2579','I25790','I25791','I25798','I25799','I258','I2581','I25810','I25811','I25812','I2582','I2583','I2584','I2589','I259' |
| Congenital heart disease | '7450','74510','74511','74512','74519','7452','7453','7454','7455','74560','74561','74569','7457','7458','7459','74600','74601','74602','74609','7461','7462','7463','7464','7465','7466','7467','74681','74682','74683','74684','74685','74686','74687','74689','7469','7470','74710','74711','74720','74721','74722','74729','7473','74731','74732','74739','74740','74741','74742','74749','7475','74760','74761','74762','74763','74764','74769','74781','74782','74783','74789','7479' | 'Q20','Q200','Q201','Q202','Q203','Q204','Q205','Q206','Q208','Q209','Q21','Q210','Q211','Q212','Q213','Q214','Q218','Q219','Q22','Q220','Q221','Q222','Q223','Q224','Q225','Q226','Q228','Q229','Q23','Q230','Q231','Q232','Q233','Q234','Q238','Q239','Q24','Q240','Q241','Q242','Q243','Q244','Q245','Q246','Q248','Q249','Q255','Q256','Q25','Q250','Q251','Q252','Q2521','Q2529','Q253','Q254','Q2540','Q2541','Q2542','Q2543','Q2544','Q2545','Q2546','Q2547','Q2548','Q2549','Q257','Q2571','Q2572','Q2579','Q258','Q259','Q26','Q260','Q261','Q262','Q263','Q264','Q265','Q266','Q268','Q269','Q27','Q270','Q271','Q272','Q273','Q2730','Q2731','Q2732','Q2733','Q2734','Q2739','Q274','Q278','Q279','Q28','Q280','Q281','Q282','Q283','Q288','Q289' |
| Other cardiomyopathies | '4250','4251','42511','42518','4252','4253','4254','4255','4257','4258','4259',41081, 41082, 41090, 41091, 41092, 412 | 'I428','I429','I42','I420','I421','I422','I423','I424','I425','I426','I427' |
| Cardiac valvulopathies | '4240','4241','4242','4243','42490','42491','42499', | 'I34','I340','I341','I342','I348','I349','I35','I350','I351','I352','I358','I359','I36','I360','I361','I362','I368','I369','I37','I370','I371','I372','I378','I379', |
| Chronic pulmonary heart disease | '4160','4161','4162','4168','4169' | 'I2722','I2723','I27','I270','I271','I272','I2720','I2721','I2724','I2729','I278','I2781','I2782','I2783','I2789','I279' |
| Rheumatic heart disease | '393','3940','3941','3942','3949','3950','3951','3952','3959','3960','3961','3962','3963','3968','3969','3970','3971','3979','3980','39890','39891','39899' | 'I06','I060','I061','I062','I068','I069','I05','I050','I051','I052','I058','I059','I07','I070','I071','I072','I078','I079','I08','I080','I081','I082','I083','I088','I089','I09','I090','I091','I092','I098','I0981','I0989','I099' |
| Clinical trial | 'V707' | 'Z006' |
| Chronic pulmonary disease | SELECT * FROM "d_icd_diagnoses"  where  icd_version= 9  AND  (SUBSTR(icd_code, 1, 3) BETWEEN '490' AND '505'  OR  SUBSTR(icd_code, 1, 4) IN ('4168','4169','5064','5081','5088')) | SELECT * FROM "d_icd_diagnoses"  where  icd_version= 10  AND  (  SUBSTR(icd_code, 1, 3) BETWEEN 'J40' AND 'J47'  OR  SUBSTR(icd_code, 1, 3) BETWEEN 'J60' AND 'J67'  OR  SUBSTR(icd_code, 1, 4) IN ('I278','I279','J684','J701','J703')  ) |
| Diabetes | SELECT * FROM "d_icd_diagnoses"  where  icd_version= 9  AND  ( SUBSTR(icd_code, 1, 4) IN ('2500','2501','2502','2503','2508','2509') )；  SELECT * FROM "d_icd_diagnoses"  where  icd_version= 9  AND  ( SUBSTR(icd_code, 1, 4) IN ('2504','2505','2506','2507') ) | SELECT * FROM "d_icd_diagnoses"  where  icd_version= 10  AND  (  SUBSTR(icd_code, 1, 4) IN ('E100','E10l','E106','E108','E109','E110','E111', 'E116','E118','E119','E120','E121','E126','E128',  'E129','E130','E131','E136','E138','E139','E140',  'E141','E146','E148','E149')  )；SELECT * FROM "d_icd_diagnoses"  where  icd_version= 10  AND  (  SUBSTR(icd_code, 1, 4) IN ('E102','E103','E104','E105','E107','E112','E113',  'E114','E115','E117','E122','E123','E124','E125',  'E127','E132','E133','E134','E135','E137','E142',  'E143','E144','E145','E147')  ) |
| Renal disease | SELECT * FROM "d_icd_diagnoses"  where  icd_version= 9  AND  ( SUBSTR(icd_code, 1, 3) IN ('582','585','586','V56')  OR  SUBSTR(icd_code, 1, 4) IN ('5880','V420','V451')  OR  SUBSTR(icd_code, 1, 4) BETWEEN '5830' AND '5837'  OR  SUBSTR(icd_code, 1, 5) IN ('40301','40311','40391','40402','40403','40412','40413','40492','40493') ) | SELECT * FROM "d_icd_diagnoses"  where  icd_version= 10  AND  (  SUBSTR(icd_code, 1, 3) IN ('N18','N19')  OR  SUBSTR(icd_code, 1, 4) IN ('I120','I131','N032','N033','N034',  'N035','N036','N037','N052','N053',  'N054','N055','N056','N057','N250',  'Z490','Z491','Z492','Z940','Z992')  ) |
| Atrial fibrillation | '42731 | 'I48,'I480,'I481,'I4811,'I4819, 'I482, 'I4820, 'I4821, 'I489, 'I4891 |
| Hypertension | '4010','4011', '4019', '40200', '40201', '40210','40211','40290','40291','40300','40301','40310','40311','40390','40391','40400','40401','40402','40403','40410','40411','40412','40413','40490','40491','40492','40493','40501','40509','40511','40519','40591', '40599', '4372', '7600', '7962', '99791', | 'I10','I11','I110','I119','I12','I120','I129','I13','I130', 'I131', 'I1310', 'I1311', 'I132', 'I15', 'I150','I151','I152','I158','I159','I16','I160','I161','I169','I674','I973','O1003','O1013','O1023', 'O1033','O1043','O1093','O16', 'O165', 'P000', 'P292', 'R030', 'V811' |

*SQL* Structured Query Language; *ICD* International Classification of Diseases
