# Supplementary material for: Development and validation of a nomogram to predict risk of septic cardiomyopathy in the intensive care unit
Source: Sci Rep. 2024 Jun 19;14:14114. doi: 10.1038/s41598-024-64965-x (PMC11187202; doi:10.1038/s41598-024-64965-x)
Supplement: Supplementary file 2 — Supplementary Table S2. [file 41598_2024_64965_MOESM2_ESM.docx]

| Variables | Number(%) with missing data | Complete case  (n = 393) | Multiple imputation (n = 1562) | P |
| --- | --- | --- | --- | --- |
| Troponin T ,ng/ml, mean (SD) | 695(44.4) | 0.112 (0.472) | 0.077 (0.367) | 0.11 |
| CK-MB index, mean (SD) | 913(58.4) | 0.041 (0.064) | 0.035 (0.098) | 0.284 |
| Lactate,mmol/L, median (IQR) | 379(24.2) | 1.70 (1.20, 2.80) | 1.68 (1.20, 2.44) | 0.141 |
| ALT,U/L, median (IQR) | 368(23.5) | 34.5 (20.0, 72.0) | 31.0 (18.5, 67.5) | 0.08 |
| AST,U/L, median (IQR) | 369(23.6) | 51.0 (29.0, 124.0) | 47.0 (27.0, 112.4) | 0.099 |
| White blood cell, ×109/L, median (IQR) | 2(0.1) | 12.1 (8.5, 17.0) | 11.5 (7.9, 16.0) | 0.063 |
| Heart rate,bpm, mean (SD) | 3(0.1) | 91.2 (18.2) | 89.6 (17.94) | 0.112 |
| SBP,mmHg, mean (SD) | 8(0.5) | 115.4 (16.0) | 117.2 (16.6) | 0.059 |
| DBP,mmHg, mean (SD) | 8(0.5) | 63.6 (10.2) | 63.3 (10.5) | 0.66 |
| MBP,mmHg, mean (SD) | 3(0.1) | 77.6 (10.5) | 77.7 (10.9) | 0.868 |
| Respiratory rate , mean (SD) | 3(0.1) | 21.1 (4.6) | 20.6 (4.5) | 0.061 |
| Temperature,℃,, mean (SD) | 21(1.9) | 36.9 (0.9) | 37.0 (0.7) | 0.261 |
| Spo2,%, mean (SD) | 5(0.3) | 96.7 (2.8) | 96.7 (2.3) | 0.933 |
| ARV of 24h heart rate, mean (SD) | 3(0.1) | 5.3 (2.7） | 5.4 (2.8) | 0.672 |
| ARV of 24h SBP, mean (SD) | 8(0.5) | 12.0 (4.9) | 11.6 (4.5) | 0.084 |
| ARV of 24h DBP, mean (SD) | 8(0.5) | 8.8 (4.6) | 8.7 (4.3) | 0.635 |
| Arv of 24h MBP, mean (SD) | 3(0.1) | 9.4 (4.0) | 9.2 (4.4) | 0.341 |
| ARV of 24h respiratory rate, mean (SD) | 3(0.1) | 3.2 (1.1) | 3.3 (1.1) | 0.132 |
| ARV of 24h temperature | 21(1.9) | 0.4 (0.1) | 0.4 (0.2) | 0.166 |
| ARV of 24h Spo2, mean (SD) | 5(0.3) | 1.5 (0.8) | 1.5 (0.70 | 0.969 |

**Table S2:** Distributions of variables with missing data comparing complete case data to results from pooling the datasets with imputed variables from multiple imputation.

*SD* Standard Deviation. *IQR* interquartile range. *SBP s*ystolic blood pressure. *DBP* diastolic blood pressure.*MBP* mean arterial pressure. *SpO2* peripheral oxygen saturation. *ARV* average real variability. *WBC* White blood cell.*BUN* blood urea nitrogen. *ALT* alanine aminotransferase. *AST* aspartate aminotransferase. *CK-MB* Creatine Kinase Isoenzyme-MB. *ICU* intensive care unit.
